# Psychiatric Manifestations of Neurological Diseases: A Narrative Review

**DOI:** 10.7759/cureus.64152

**Published:** 2024-07-09

**Authors:** Anthony J Maristany, Brianna C Sa, Cameron Murray, Ashwin B Subramaniam, Sean E Oldak

**Affiliations:** 1 Psychiatry and Behavioral Sciences, University of Miami Leonard M. Miller School of Medicine, Miami, USA; 2 Psychiatry and Behavioral Sciences, University of Miami Leonard M. Miller School of Medicine/Jackson Memorial Hospital, Miami, USA

**Keywords:** mental health issues, psychiatry and neuroscience, psychiatry & mental health, psychiatric manifestations, neurologic disorders

## Abstract

Neurological diseases often manifest with psychiatric symptoms, profoundly impacting patients' well-being and treatment outcomes. This comprehensive review examines the psychiatric manifestations associated with Alzheimer's disease, frontotemporal dementia (FTD), Parkinson's disease, multiple sclerosis (MS), stroke, epilepsy, Huntington's disease, amyotrophic lateral sclerosis (ALS), traumatic brain injury (TBI), and multiple system atrophy (MSA). Key psychiatric symptoms include agitation, depression, anxiety, apathy, hallucinations, impulsivity, and aggression across these diseases. In addition, ethical considerations in treating these symptoms are paramount, particularly regarding genetic testing implications, end-of-life discussions, informed consent, and equitable access to innovative treatments. Effective management necessitates interdisciplinary collaboration, personalized interventions, and a focus on patient autonomy. Understanding the psychiatric burden of neurological diseases is crucial for enhancing patients' quality of life. Further research is needed to elucidate underlying mechanisms and develop targeted interventions. This review underscores the importance of comprehensive assessment and ethical treatment practices to address psychiatric manifestations effectively.

## Introduction and background

Though once considered disparate disciplines, it has become more and more evident, as our understanding of the brain continues to expand, that psychiatric and neurologic diseases are decidedly interconnected. Neurologic diseases, which are characterized by disruptions in neuronal circuitry, often are associated with psychiatric symptomatology that may challenge conventional diagnostic boundaries and necessitate the need for more comprehensive approaches to patient care. Understanding the impact of psychiatric manifestations on patients, their families, and caregivers is paramount in providing effective and empathetic care.

This narrative review seeks to delve into the various psychiatric manifestations of neurologic disease and the intricate interplay between neurological and psychiatric diseases. We review a spectrum of neurological disorders and underscore the various psychiatric symptoms often associated with them, what is known about the neurobiologic mechanisms of these symptoms in the specific neurological disorders, as well as review diagnostic challenges, innovative treatment approaches, ethical considerations, and future research directions. By delving into the underlying neurobiological mechanisms driving these manifestations, we seek to uncover both commonalities and unique features across different diseases. Moreover, our objective extends beyond mere exploration; we intend to discuss innovative treatment strategies and outline potential future research directions in this multifaceted field. 

## Review

Methods

The literature search for the review was conducted using online databases including PubMed, Google Scholar, and the National Center for Biotechnology Information (NCBI) database. The exclusion criteria included studies published before 1990. Keywords used in search engines included: psychiatric manifestations in Alzheimer's disease (AD), psychiatric manifestations in frontotemporal dementia (FTD), psychiatric manifestations in Parkinson's disease, psychiatric manifestations in multiple sclerosis (MS), psychiatric manifestations in stroke, psychiatric manifestations in epilepsy, psychiatric manifestations in Huntington's disease, psychiatric manifestations in amyotrophic lateral sclerosis (ALS), psychiatric manifestations in traumatic brain injury (TBI), and psychiatric manifestations in multiple system atrophy (MSA)

Neurological diseases and their psychiatric manifestations

This review examines the psychiatric manifestations associated with AD, FTD, Parkinson's disease, MS, stroke, epilepsy, Huntington's disease, ALS, TBI, and MSA. These diseases were chosen for their high prevalence, complex etiologies, and comorbid psychiatric symptomatology.

AD

AD is characterized by a slow rate of cognitive decline ultimately leading to a type of dementia that impacts memory, behavior, and reasoning. On average, global cognitive decline begins 7.5 years prior to the diagnosis of dementia and significantly accelerates 5.5 years after the onset of symptoms [1]. The pathophysiology behind AD involves the aggregation of amyloid β plaques and tau protein-derived neurofibrillary tangles which have been directly associated with many of the hallmark neuropsychiatric symptoms commonly observed in Alzheimer’s patients [2]. 

Early symptoms of Alzheimer’s include, but are not limited to, difficulty recalling specific events, remembering names, and misplacing valued items [2]. Most often, memory impairment worsens and is accompanied by frequent expressions of emotional distress and sudden personality changes [2]. Patients often lose the ability to carry out basic activities of daily living (ADLs) such as brushing their teeth, writing, and reading without assistance. Despite becoming dependent on others, Alzheimer’s patients experience personality changes that often manifest in the form of increased agitation, inclusiveness, and apathy [2]. The development of these behaviors can make it difficult for these patients to maintain relationships with friends and family thereby impeding the likelihood of these patients receiving the assistance they need. 

Alzheimer’s patients experience neuropsychiatric symptoms at all stages throughout the progression of the disease, with certain symptoms appearing and predominating at various points along its progression. Agitation and aggression are frequently observed at all stages of the patient’s cognitive decline and, like other symptoms, correlate with the progression of cognitive decline [3]. Few studies have been done on the prevalence of agitation and aggression as symptoms of AD; however, one study on a large sample of electronic medical records showed that approximately 50% of mild Alzheimer’s patients displayed signs of agitation [3]. Agitation and aggression in AD have been specifically associated with a wide array of alleles including those responsible for Aβ42 protein expression and numerous serotonin transporter polymorphisms [4]. Agitation and aggression are some of the greatest contributors to the cost of care with many Alzheimer’s patients being admitted to institutions and requiring greater caretaker supervision [3].

Apathy and depression often occur comorbidly with depression being one of the most common symptoms in the early stages of AD [3]. While depression tends to stabilize during the course of cognitive decline, apathy often progresses. In a large cohort study of 255 Alzheimer’s patients, 48% had depression, 42% had apathy, and 32% had both [5]. Reported rates of depression in Alzheimer’s studies vary significantly (25-75%) due to the many existing methods of diagnosis [4,6]. Numerous studies have shown that AD with comorbid depression and/or apathy results in a poorer prognosis [4]. The pathogenesis of AD has been linked to the development of both apathy and depression with neurodegeneration of specific brain areas increasing the risk of developing these psychiatric symptoms [4]. For example, in Alzheimer’s patients, depression is associated with hypometabolism of the left dorsolateral prefrontal areas while apathy is associated with hypometabolism of the left orbitofrontal areas [4]. Numerous studies have looked at certain genetic risk factors for depression in AD that are related to the pathogenesis of the disease including *APOE4* and the CC genotype of transforming growth factor-β1 (TGF-β1), +10 T/C single nucleotide polymorphism (SNP). While studies on *APOE4* showed mixed results, a five-fold increase in depression was observed among Alzheimer’s patients who expressed the CC genotype of TGF-β1 +10 T/C SNP [4]. Interestingly, recent studies have potentially identified certain genetic risk factors for the development of depression in AD but not AD without depression including the tryptophan hydroxylase-1 (TPH1) A218C allele, monoamine oxidase A (MAOA) variable number tandem repeat (VNTR), and BDNF Val66Met allele [4]. Further similar studies with respect to apathy are needed.

Major sleep problems are another psychiatric symptom of Alzheimer’s with up to 45% of patients experiencing sleep disturbances [7]. While aging can naturally result in an increase in sleep disturbances, those experienced by Alzheimer’s patients are more severe. Research suggests that this heightened severity of sleep disturbances is likely due to the increased wakefulness after sleep onset and increased sleep latency associated with the disease [7]. 

The prevalence of hallucinations in Alzheimer’s patients ranges from 12% to 33% although they are less common than other psychiatric symptoms in the disease’s early stages [3,8]. Hallucinations have been largely shown to be associated with a higher rate of cognitive decline, although studies show conflicting results about their role as predictors of disease severity [3]. Hallucinations are more likely to occur in Alzheimer’s patients as they are falling asleep and during sleep-wake transitions which plays a significant role in contributing to the major sleep problems these patients face [3]. 

It is important to note the synergistic effects each of these symptoms can have on one another. Sleeping problems and extensive sleeping during the day result in increased levels of isolation and detrimental effects on cognition. Isolation has been shown to increase the likelihood of developing depression which can lead to higher levels of apathy and emotional stress [6]. Research on the various clusters of neuropsychiatric symptoms in Alzheimer’s patients is needed before new treatments can be developed to slow or break this positive feedback loop of psychiatric symptoms.

FTD 

FTD is a subtype of dementia that typically develops in middle to late-age adults and encompasses three subtypes of disorders that are categorized by different clinical presentations [9]. Early hallmark symptoms of FTD include behavioral and language manifestations, a feature that may help to distinguish it from AD which often begins with memory loss [9]. FTD often involves genetic factors, with mutations in the *C9orf72* gene being the most common, responsible for 25% of familial FTD cases. Psychotic symptoms are particularly common in individuals with mutations in either the progranulin or *C9orf72* genes. Genetic testing can be useful in managing the severity of this disease [9].

The rarest form of FTD involves degeneration of frontal and temporal lobes and may also include behavioral and or language symptoms. This form includes disorders that fall under the same syndrome such as corticobasal syndrome, progressive supranuclear palsy, FTD with parkinsonism, and FTD with amyotrophic lateral sclerosis [9]. The second most common form of FTD is Primary Progressive Aphasia (PPA) which is characterized by the gradual deterioration of one’s ability to communicate [9]. Patients with PPA initially present different symptoms related to communication which are used to further categorize their form of FTD [9]. Behavioral FTD (bvFTD) is the most common variant and includes symptoms such as heightened impulsivity, lack of social awareness, agitation, and poor judgment [10]. People with bvFTD tend to exhibit strong sudden changes to their personalities which often result in an increased tendency to commit social transgressions. FTD is a generally slow-progressing disease that often includes the development of sociopathic and psychotic symptoms. Diagnosing FTD at any stage of life has proven to be difficult due to overlapping symptoms with various psychiatric disorders [10].

There have been several cases of individuals with bvFTD engaging in sociopathic and criminal behavior as a result of their condition. This can be rather challenging in a legal context since clinical diagnosis of FTD relies on criteria like progressive personality changes, impaired social and personal conduct regulation, emotional blunting, and loss of insight [11]. Interestingly, these individuals are aware of the wrongdoings they are committing but are unable to restrain themselves from such actions due to their disease and there have been reports of individuals with this disorder who have engaged in pedophilia, theft, sexual harassment, and automobile violations despite their awareness that these actions were wrong [11]. This acquired sociopathy is associated with lesions in the ventromedial prefrontal cortex (vmPFC), the region of the brain associated with emotional processing, decision-making, and social cognition, which can cause reduced emotional responses to moral violations but possess logical reasoning and knowledge of social norms [11].

Historically, there has only been one reported case of one individual with suspected bvFTD who has engaged in violent behavior [12]. Individuals with bvFTD may commit criminal acts due to inadequate control over instinctive urges. However, they seldom exhibit aggressive behavior leading to homicide, as emotional blunting in bvFTD reduces impulsive aggression triggers [12]. Legal dispositions in psychiatric-legal cases should account for these nuances for optimal understanding [13]. Case-by-case evaluations, considering neurobiological deficits and cognitive capacity, are crucial when individuals with bvFTD are involved in criminal legal proceedings [12].

Parkinson's Disease 

Parkinson's disease is a neurodegenerative disorder that is predominantly characterized by motor dysfunction that it inflicts amongst afflicted patients [14,15]. Moreover, it is characterized by the progressive degeneration of dopaminergic neurons originating predominantly in the substantia nigra pars compacta, and these neurons help modulate simple and complex movements [15]. Parkinson's disease has also been implicated in potentially causing or exacerbating various psychiatric conditions, including depression, anxiety, apathy, psychosis, and impulse control disorders [16]. The prevalence of depression among Parkinson's patients has been estimated to be between 30% and 35% [15]. There are multiple neuronal systems involved in Parkinson’s disease-associated depression [15]. Specifically, decreased dopamine transporter concentration at the limbic and striatal regions, reduced acetylcholine expression, decreased forebrain serotonin activation, and increased neuronal gliosis and loss in the locus coeruleus have all been identified as possible pathophysiological causes associated with depression manifestation amongst patients with Parkinson's disease [16]. 

Additionally, the prevalence of anxiety among patients with Parkinson's disease has been estimated to be at 31%, which is higher than other diseases that cause similar dysfunction [15]. The pathophysiology of Parkinson’s associated anxiety has not been well elucidated, but there is evidence that the elimination of subcortical nuclei and ascending dopamine pathways, as well as reductions in the sizes of the amygdala, anterior cingulate cortex, and orbitofrontal cortex, could play some role in anxiety presentation [16]. Patients can present with a mixture of somatic and behavioral symptoms, including palpitations, shortness of breath, digestive upset, inability to relax, and feeling perennially tense and restless [15]. Standard treatments used in anxiety management, such as the prescription of selective serotonin reuptake inhibitors (SSRIs), benzodiazepines, and buspirone, can be used when addressing anxiety-associated symptoms among patients with Parkinson's disease [16].

Apathy and psychosis have also been associated with some Parkinson’s patients. Apathy can be described as a marked loss of motivation that cannot be attributed to emotional or intellectual impairment or a loss of consciousness [15]. The prevalence of apathy among patients with Parkinson's is between 16.5% and 40% [16]. The severity of motor symptoms, due to decreases in dopamine concentrations in the nigrostriatal pathway, has been shown to serve as a possible proxy for apathy [16]. Moreover, atrophy of the left nucleus accumbens and reductions of gray matter density in the cingulate and inferior frontal gyri may induce rapid apathy onset [16]. On the other hand, psychosis typically presents amongst patients with Parkinson's through visual hallucinations, illusions, and delusions [15]. There is a wide range of Parkinson’s disease-associated psychosis prevalences reported, with estimates of prevalences between 26% and 82.7% [16]. The pathophysiology of psychosis presentation has not been sufficiently explored, but atrophy of the dorsal and ventral visual stream provides early insight into possible causes [15]. Other possible explanations include a decrease in retinal dopamine concentration, Lewy body accumulation in the amygdala and parahippocampal gyrus, and noticeable formation of amyloid and tau plaques in the frontal, hippocampus, and parietal regions [14].

Impulse control disorders may also be seen among Parkinson’s patients, and these disorders are characterized by behaviors performed repetitively, excessively, and compulsively which then pose significant emotional and physical distress in the daily lives of patients [15]. The reported prevalence of these disorders in Parkinson’s patients varies between 35.9% and 60% [16]. Dopamine replacement therapy has been shown to serve as a significant risk factor in impulse control exacerbation amongst patients with Parkinson's [16]. Hyperactivity and hypoactivity of the ventral striatum interestingly have both been observed amongst patients undergoing dopamine replacement therapy [16]. Additionally, reduced activity in the lateral orbitofrontal cortex and rostral cingulate has been noted among patients taking dopamine agonists [16]. The risks of developing impulse control disorders are further heightened if such agonists, such as pramipexole and rotigotine, are preferentially directed towards the D2 and D3 receptors, hinting that both the mesocortical and mesolimbic pathways are involved [16]. To this date, there are no treatments that successfully eliminate impulse control disorders, but there are clinical trials currently being conducted with various medication classes to address these problems [15]. 

There are several ethical considerations to account for when treating patients with comorbid psychiatric disorders and Parkinson’s disease, including communication skills, assisting patients with coping with Parkinson’s, identity assessment, and planning future medical treatment [17]. With regard to proper communication, it is important for physicians to carefully assess the patient’s preferences for how they would like their relevant medical information and results to be presented to them [17]. It is also important that physicians deliver all relevant updates with compassion and care. When coping with Parkinson’s, patients may request physicians to collaborate closely with other members of the healthcare team to ensure that their requests are being met [17]. When assessing how patients feel about themselves, it seems that the loss of independence with respect to motor function can result in feelings of worthlessness. Consequently, physicians are advised to listen and let patients share how they feel to provide them with a sense of autonomy [17]. Conversations with patients about future medical treatment pose challenges for physicians, as they may be reluctant to share with patients the possibility of severe cognitive decline and dysfunction [17]. Further research should be conducted to investigate how to best address such concerns appropriately with patients while taking their desires into consideration.

MS 

MS is a complex autoimmune disease characterized by chronic inflammation and demyelination within the central nervous system. MS is relatively common, impacting approximately 2.3 million individuals worldwide, with a higher prevalence among young females [18]. MS typically presents as a chronic relapsing and remitting disease course, characterized by alternating periods of neurologic symptom exacerbation and remission. This condition leads to various physical debilitations, including cognitive symptoms, vision loss, muscle weakness, fatigue, and pain, all of which contribute to mobility challenges. Moreover, MS often results in autonomic dysfunction, encompassing issues like bladder and sexual dysfunction [19]. The cumulative effect of these factors has a substantial impact on the patient's overall quality of life. This interplay among physical symptoms, cognitive dysfunction, and emotional disturbances may help account for the high prevalence of psychiatric conditions in MS patients, affecting nearly two-thirds of individuals with the condition [19].

Fatigue is an overwhelmingly prevalent and debilitating symptom in MS, affecting up to 85% of patients, and is considered the most debilitating aspect of MS by 60% of patients [18]. MS-related fatigue is not merely a consequence of physical exhaustion; rather, it arises from a complex interplay of factors encompassing disruptions in immune responses, hormonal pathways, and neural networks associated with motor preparation, including the fronto-thalamic pathways and basal ganglia [18]. Mobility challenges, from dysregulated motor control to fatigue and pain, which are prevalent in two-thirds of MS patients, can impair a patient's functionality and can evoke feelings of frustration, discontentment, and powerlessness [18,20]. Patients facing these challenges often struggle to fulfill their usual roles and enjoy activities they once found pleasurable, further laying the foundation for mood disturbances. In this context, resilience and positive coping strategies become vital protective factors against the development of depression in MS [18]. Cognitive reframing, for instance, can help individuals positively reframe their challenges, promoting a more adaptive perspective. On the other hand, maladaptive coping strategies, such as seeking emotional respite or escape avoidance, are strong predictors of depression development in the MS population [18]. 

Conversely, mood disturbances, particularly depression, can exacerbate fatigue in individuals with MS. Depression is the most common psychiatric condition in MS, affecting 40-50% of patients during their lifetime [19]. In comparison to the general population, MS patients face a two to five times higher risk of depression, and this risk surpasses that of individuals with other chronic illnesses as well [18,19]. Notably, depression is the main predictor of poor quality of life in MS patients and depressive symptoms are reported to increase in the month preceding and during an MS relapse [21]. Depression can significantly disrupt sleep patterns, alter neurotransmitter levels, and foster consumptive negative thought patterns, all of which contribute to the experience of fatigue [21,22]. When it comes to patients with MS, this complex interaction raises an intriguing question: Is a patient's fatigue primarily a manifestation of the disease, a result of depression, or a combination of both?

It is important to note that there might be shared underlying mechanisms between the physical symptoms experienced by individuals with MS and the mood disturbances that can accompany the condition. In MS, the presence of white matter lesions on neuroimaging is a hallmark of the disease and represents focal areas of demyelination [18-20]. However, in MS patients who also experience depression, a distinct pattern of demyelination and imaging findings are present. More precisely, individuals with both MS and depression tend to display a higher burden of white matter lesions in the prefrontal cortex [21]. This increased lesion load is associated with the severity of depressive symptoms and contributes to a reduction in the volume of white matter in the frontal cortex [21]. Furthermore, depressed individuals with MS exhibit compromised neural fiber integrity in regions responsible for regulating connections between the frontal and limbic areas, possibly fostering emotional dysregulation. Additionally, in individuals with both MS and depression, gray matter lesions cause cortical thinning. Thinning of the frontal cortex predicts MS-related depression, while reduced thickness in the right entorhinal cortex is linked to the severity of depressive symptoms [21]. Treatment of depression in MS abides by the same guidelines as patients without MS, treatment should initiate with SSRIs or serotonin and norepinephrine reuptake inhibitors (SNRIs), targeting therapy to avoid drug interactions or minimize adverse effects [18]. For the treatment of fatigue in MS patients who do not experience depression, amantadine, an N-methyl-D-aspartate (NMDA) antagonist, and modafinil, a narcolepsy agent, which both have dopaminergic effects, are typically first-line [18]. 

Cognitive dysfunction is also a prominent feature of MS, affecting more than half of patients, particularly within the first five years of diagnosis [20]. The impairments can range from slowed information processing, conceptual reasoning, attention, memory, and visuospatial malfunction [20]. Cognitive decline in MS is associated with various risk factors including disease duration, male gender, genetics, and disability level [18]. In addition to the aforementioned risk factors, certain clinical findings can also provide valuable insights into cognitive impairment in MS patients. Notably, patients with subcortical involvement, particularly within the corpus callosum, are more likely to have slower processing speeds, attention difficulties, memory dysfunction, and impaired abstract reasoning [18,19]. Additionally, microstructural changes of white matter on MRI and the extent of neuronal, specifically thalamic, atrophy serve as predictors of cognitive decline [18]. It is also worth emphasizing that depression, as previously mentioned, can significantly exacerbate cognitive dysfunction in MS patients. The cognitive domains most commonly impacted in MS include processing speed, attention, and working memory, often associated with frontal and parietal lobe lesions. A large percentage of patients will also suffer from impaired visuospatial perception and abilities. Comprehensive language skills tend to remain intact, although some patients may suffer from compromised word retrieval [18]. All MS patients should have regular screening for and assessment of cognitive impairment to prevent progression and optimize patient outcomes [23]. Disease-modifying treatment for MS is imperative in preventing the development of and progression of cognitive impairment [23].

Stroke 

A stroke is a cerebrovascular accident caused by either a thrombus or hemorrhage that leads to ischemia of brain parenchyma. Strokes are profoundly traumatic for patients, causing both physiological and psychological distress. In the post-stroke phase, a multitude of psychiatric manifestations arise, each rooted in various etiologies. Focal neurological impairments post stroke have substantial emotional ramifications for patients. Motor impairments such as hemiparesis or fatigue, can compromise the patient’s independence, requiring assistance with ADLs or inhibiting them from participating in enjoyable activities [24]. Additionally, deficits such as cognitive impairment and aphasia, can evoke feelings of frustration and isolation from an inability or decreased ability to communicate and socialize. Such life changes and insult to neuronal function lay the foundation for the development of psychiatric disease in the post-stroke period.

Post-stroke depression affects 20% of stroke patients [25] and is associated with long-term negative effects on social functioning, quality of life, and short-term mortality [24,25]. The pathophysiology of poststroke depression is complex and not fully elucidated, but it may involve genetic predisposition with modifications to serotonin transporter genes, hypothalamic-pituitary-adrenal (HPA) axis-induced hypercortisolemia, proinflammatory cytokines, and glutamate-mediated excitotoxicity [26,27]. Risk factors for post-stroke apathy and depression include severity of stroke, history of cerebrovascular disease, hypertension, and severe cognitive impairment [26]. Post-stroke depression patients are more likely to suffer from multiple infarcts, large lesions, and left hemispheric infarcts, specifically those of the prefrontal subcortical circuits [27]. Post-stroke depression can occur up to 18 months after the event and is typically screened for with the Patient Health Questionnaire-9 (PHQ-9) assessment, which has high sensitivity and specificity [26]. Screening can be complicated as neurological sequelae of strokes such as anosognosia, cognitive impairment, and aprosodia can mimic symptoms of depression and vice versa [26]. Early identification and treatment are pivotal to improving patient outcomes [25]. Treatment with SSRIs, tricyclic antidepressants (TCAs), or electroconvulsive therapy (ECT) has been shown to be efficacious, keeping in mind patient-specific factors and the risk for hemorrhage [25]. 

Post-stroke anxiety affects up to 24% of stroke patients [26] and has been found to often coincide with post-stroke depression [25]. Risk factors for developing post-stroke depression include genomic polymorphisms in serotonin transporters, and suffering from chronic pain, sleep disturbances, and communication challenges [26,28]. There may be an association between cerebral hemispheric white matter strokes and the development of post-stroke depression, but the evidence needs to be corroborated and further studied [28,29]. The long-term quality of life for patients with post-stroke anxiety is poor [28]. Treatment may include SSRIs, SNRIs, TCAs, and non-pharmacologic interventions such as cognitive therapy [24]. Studies evaluating risk factors for and treatment efficacy in post-stroke anxiety should be completed to better understand this condition. 

Epilepsy 

Seizures impose a considerable emotional toll on patients living with epilepsy. Seizures manifest in diverse forms, from absence seizures consisting of “zoning out” episodes to generalized tonic-clonic seizures consisting of full-body rhythmic convulsions, and each presents with its own set of challenges. Seizures in epilepsy are unpredictable and this uncertain nature can cause severe anxiety and fear for future events in patients. Psychiatric manifestations develop in 50-60% of patients with epilepsy, most often mood, anxiety, and psychotic disorders [30]. Psychiatric manifestations can be preictal, ictal, postictal, or interictal [30], with most patients experiencing ictal manifestations [31]. Seizure-related, ictal, psychiatric manifestations encompass anxiety, intense fear, panic attacks, paranoia, confusion, depressed mood, and aggression. Post-ictal manifestations share similarities with ictal manifestations but anxiety or fear and feature significant confusion. Interictally, patients may exhibit various psychiatric disorders, most commonly mood disorders [30]. There is a bidirectional relationship between epilepsy and mood disorders, such as depression and anxiety. Patients with epilepsy may develop mood disorders as a consequence of their condition, ranging from medication side effects to trauma and encephalitis [31]. Conversely, mood disorders can exacerbate the frequency and severity of seizures [31]. 

Over 50% of epilepsy patients develop major depressive disorder (MDD) interictally [30]. Risk factors for the development of depression in epilepsy include left hemisphere foci, frontal and temporal lobe dysfunction, duration and severity of epilepsy, perceived stigma of the disease, decreased social support, learned helplessness, and polytherapy with antiepileptic drugs. Diagnosis and management of depression in epilepsy patients is paramount as suicide is four times more common in epilepsy patients when compared to the general population. Risk factors for suicidality in epilepsy patients include prior suicide attempts, substance abuse, personal or familial psychiatric disorders, and surgically treated epilepsy [30]. Localization of epilepsy may mediate the psychiatric manifestations patients experience. Generalized tonic-clonic epilepsy, which presents with whole-body rhythmic jerking, is most frequently associated with confusion and cognitive impairment [32]. Temporal lobe epilepsy presents with focal seizures which last one to three minutes associated with auras and oroalimentary automatisms [31]. Temporal lobe epilepsy, the most common type of focal epilepsy, predisposes patients to memory deficits, cognitive impairment, depression, anxiety, schizophrenia, and paranoid hallucinatory psychotic symptoms [30,32]. Interestingly in one-third of patients with temporal lobe epilepsy, the severity of seizures is inversely related to psychiatric symptoms [32]. Additionally, in these patients, as seizures electrically stabilize on EEG, psychiatric symptoms emerge also known as “forced normalization” [32]. Frontal lobe epilepsy, the second most common focal epilepsy, is characterized by brief nocturnal seizures with proximal automatisms [31]. Frontal lobe epilepsy can present with cognitive changes, decreased social awareness, sleep disruption, “frontal syndrome” involving sexual disinhibition, loss of concern for personal hygiene, aggression, depression, and altered speech patterns. In pediatric frontal lobe epilepsy patients, 76% have comorbid attention deficit hyperactivity disorder (ADHD) [31].

Psychiatric comorbidities among individuals with epilepsy significantly influence seizure control management. Treatment depends on the timing of psychiatric symptoms, whether periictal or interictal. The first-line treatment for ictal psychiatric manifestations is seizure control with antiepileptic medications, as symptoms might resolve with the management of epilepsy [31]. If seizure management doesn’t adequately control the psychiatric symptoms then psychotropic medications may be added [31]. Notably, some antiepileptic medications have mood-stabilizing or antipsychotic effects including valproic acid, carbamazepine, and lamotrigine [31]. Conversely, certain antiepileptic drugs like topiramate, vigabatrin, and levetiracetam can exacerbate psychiatric manifestations and should be avoided in comorbid epilepsy and psychiatric conditions [31]. For interictal management, typical psychotropic medications such as SSRIs, antipsychotics, and mood stabilizers are generally considered safe in epilepsy. However, caution is advised due to potential side effects, including medication interactions and alterations in seizure threshold, as seen with drugs like bupropion [31,33].

Huntington's Disease 

Huntington's disease is an autosomal dominant neurological disorder that commonly affects individuals starting at the age of 40 years [34]. Patients usually present with a variety of motor-related dysfunctions, including but not limited to bradykinesia, chorea, and dystonia [34]. However, patients will also present with several psychiatric-related problems that can induce significant emotional and physical distress. Some of these include altered personality changes, depression, and heightened agitation or irritability. Patients have also been hospitalized for expressing symptoms associated with psychosis, such as grandiose delusions and sensory hallucinations [34,35]. The association between chorea presentation and emotional distress has increasingly become tenuous, and recent studies suggest that examining cognition, functional capacity, and other motor impairments may provide better insight into the severity of psychiatric problems implicated in Huntington's disease [33]. There is scant literature present that addresses potential treatment regimens for these complex neuropsychiatric ailments, so patients are prescribed medications that are normally given to address each individual psychiatric disturbance they experience [34]. 

Among patients with Huntington's disease, dysfunction of the orbitofrontal circuit has been implicated in the onset of depression [36]. When combined with dysfunction in the medial prefrontal circuit, classic signs of depression, including increased apathy and diminished affect, can be witnessed. Patients can also present with worsened apathy through diminished function of their medial prefrontal circuit, which sends signals to the thalamus and plays a role in regulating response inhibition [36]. In order to address these symptoms, patients are prescribed SSRIs, which restore serotonin balance and facilitate effective communication between neurons [34]. Amongst patients who express apathy without depression, they may be prescribed a SNRI or an appropriate dosage of a stimulant [34]. 

Heightened aggression and irritability have also been long associated with dysfunction in the prefrontal cortex [36]. Several prefrontal regions project their neurons within the dorsolateral-subcortical circuit, which is responsible for mediating various processes including anticipatory behaviors, working memory, and attentional load [36]. Moreover, the prefrontal cortex helps modulate impulse and inhibitory control. In order to address these executive function deficits, physicians will either prescribe benzodiazepines or atypical antipsychotics [34]. The atypical antipsychotics are usually preferred because they are less likely to exacerbate existing motor deficits [34]. 

The predominant ethical challenges surrounding Huntington’s Disease stem from the available genetic testing that can be done to examine whether individuals possess the genetic abnormalities indicative of the disease [35]. Physicians are advised to work alongside genetic counselors, medical ethicists, and medical geneticists in order to determine the best course of action for the patients that they are working with. Ethical principles associated with patient autonomy, beneficence, justice, and nonmaleficence are all carefully considered when discussing how to proceed forward with genetic testing. Moreover, physicians should evaluate who will benefit if such medical information is disseminated and who may be harmed. If the patient proceeds to have children who are at high risk of presenting with Huntington’s disease, the patient should discuss the logistics concerning when and how to reveal such news with their physician and a genetic counselor [35].

ALS

ALS is a neurodegenerative disorder that is best known for affecting both upper and lower motor neurons [37]. Patients diagnosed with ALS typically present with exacerbating muscle paralysis throughout their body, leading to significant functional disability, and the neurobiological mechanisms behind ALS involve the agglutination of TAR-DNA binding protein 43 (TDP43) in both types of motor neurons and the glial cells [38]. However, ALS also presents with a variety of different psychiatric symptoms, most notably affecting cognitive function, executive function, and emotional stability, which will be explored in the following sections.

There are a variety of different processes associated with executive function that are implicated in ALS. Some of these include cognitive flexibility and control, initiation and shifting of attentional resources, and verbal fluency [38]. Executive function processes are primarily implicated in the prefrontal cortex, but there hasn't been extensive research conducted examining the interplay between ALS progression and these deficits. Additionally, cognitive impairments that are often noted amongst patients with ALS include memory deficits, problems with syntactic processing, verb naming and action verb processing, and social cognition [38]. 

There are also various psychiatric problems that patients with ALS may present with. Patients may display heightened aggression and irritability, apathy, disinhibition, and rigid thinking [37]. Additionally, patients are at various risks of developing depression. According to recent literature, the incidence range is between 0.9% and 75%, and patients are more likely to develop depression within their first year. Focusing on apathy, 67% of patients with ALS have been implicated with this affective disorder. Patients may exhibit different forms of apathy, including initiation apathy (lacking drive to begin relevant actions and formulate thoughts) and emotional apathy (generalized emotional flattening and indifference) [37]. Suicidal ideation is also heightened amongst patients with ALS; specifically, patients are five to six times higher at risk than the general population [39]. These thoughts can be attributed to circumstances associated with financial instability and feeling burdensome [39]. Patients may present with anxiety and neuroticism, but these symptoms do not appear at the same frequency as the aforementioned symptoms [39]. 

With regard to caregivers and family members, behavioral and cognitive impairments pose the greatest challenges to deal with [38]. If patients are displaying increased aggression, irritability, or sadness, caregivers can themselves experience heightened stress and anxiety. Moreover, repeated visits to the doctor's office, heightened severity of symptoms, and worsened quality of life for older individuals have been shown to prolong feelings of despair amongst caregivers [38]. 

Current medication-based modalities for ALS include one of the following three medications: riluzole, edaravone, sodium phenylbutyrate, and taurursodiol [40,41]]. Riluzole, the first of these medications to be approved, mitigates glutamate release by directly blocking sodium channels [41]. Additionally, administration of riluzole assists with glutamate reuptake through activation of the transporter known as excitatory amino acid transporter 2 (EAAT2), with postsynaptic glutamate receptors also inhibited noncompetitively. These include aminomethylphosphonic acid (AMPA) and NMDA receptors. Patients taking riluzole may experience prolonged life spans of up to 19 months, and they are also able to tolerate the medication well without experiencing any adverse side effects [41]. Edavarone was the next medication to be approved, and it dampens the neuroinflammatory response and associated oxidative stress by scavenging free radicals. Patients taking this medication may experience bruising, gait abnormalities, headaches, and skin ailments. Additionally, research is underway to develop ingestible forms of edavarone for patients. The combination of sodium phenylbutyrate and taurursodiol, administered as an oral suspension, has been found to ameliorate neuronal apoptosis by reducing stress in the endoplasmic reticulum and mitochondria. Patients taking this medication primarily experience gastrointestinal-related side effects, including abdominal pain, diarrhea, and nausea. Moreover, this medication yielded survival benefits for up to 6.5 months [41]. 

Supportive care also factors heavily into treating patients with ALS. Common forms of supportive care include nutritional therapy, hospice/palliative care, physical and occupational therapy, psychotherapy, and speech therapy [41]. Healthcare providers caring for patients with ALS must remain cognizant of the many ethical challenges that exist. End-of-life conversations with patients should be started earlier than later, and it is recommended that providers adopt an approach that allows for the patient’s desires and wishes to be addressed, discussed, and accompanied [40]. Consequently, healthcare providers should avoid viewing the patient’s concerns from the perspective of change and modification [40]. Approaching patients with compassion, humility, and an understanding to listen and validate the patient’s feelings and thoughts can help providers address patient needs to the best of their abilities.

TBI 

TBI is currently the leading cause of death in young people. An estimated 1.6 million people in the United States sustain TBI per year. Due to the high degree of variability in the amount of damage and the location of the injury, a grading scale is used to determine the severity of a TBI based on the state of the patient’s consciousness. Clinical tests like the Glasgow coma scale are used to determine whether an injury is mild, moderate, or severe [42].

The likelihood of a TBI patient experiencing neuropsychiatric sequelae is highly dependent on the recency of the injury. Nearly all TBI patients experience some cognitive and/or behavioral symptoms during the acute phase of the injury, while an estimated 30-80% of those suffering from a mild to moderate injury continue to experience symptoms for up to three months. Beyond three months, approximately 15% of mild TBI patients present with enduring symptoms such as new deficits in attention, executive function, and memory [43]. Each year, approximately 85,000 TBI patients in the United States develop permanent neuropsychiatric sequelae including mania, psychosis, depression, aggression, and anxiety [43].

The most commonly observed psychiatric disorders and symptoms in TBI patients are depressive disorders, anxiety disorders, aggression, and post-traumatic stress disorder (PTSD). Currently, it is estimated that approximately 24% of patients develop an anxiety disorder following a mild TBI [43]. MDD is perhaps the most common psychiatric disorder associated with TBI and while a majority of the studies support this association, differences and flaws in methodology have produced a wide range of results. However, a more recent and promising cohort study by Bombardier et al. showed that over 53% of study participants who had suffered a TBI met the criteria for MDD at some point within 12 months post injury. After adjusting for the presence of pre-injury depression, a very high prevalence of 41% (six times higher than that of the general population) was observed which indicates a strong likelihood of developing MDD following a TBI [44]. Heightened aggression is another commonly observed symptom in TBI patients. Approximately 34% of moderate-to-severe TBI patients display relatively high levels of aggression within six months of their injury [44]. Given the association between the frontal lobe and aggressive behavior, it is conceivable that this likelihood is even higher in those whose injuries resulted in damage to the frontal lobe. The various studies on the relationship between PTSD and TBI have shown the prevalence of PTSD in TBI cases to be 10-19%, with one study reporting a prevalence of nearly 44% [44]. It is interesting to note that while a positive association has generally been observed between the severity of the TBI and the prevalence of the aforementioned psychiatric symptoms/disorders, some evidence suggests that the potential memory loss and loss of consciousness associated with severe TBIs may serve as protective factors against the development of PTSD [45]. 

Many challenges complicate the process of diagnosing TBI patients with new or worsened psychiatric symptoms. The spectrum of cognitive and behavioral changes in TBI patients who suffer from them varies drastically depending on the location and severity of the injury. More research is needed to elucidate the potential relationships between specific injury locations and associated cognitive and behavioral changes. Furthermore, neuropsychiatric symptoms and syndromes in TBI patients often diverge from our conventional understanding of them. Creating effective treatment strategies under this level of variability has proven to be difficult. Current methods of treatment often involve using a single drug to treat multiple symptoms, generally deviating from the convention of diagnosis-specific treatment strategies [46]. Pre-existing psychiatric disorders have been shown to be a significant risk factor for TBI and it has been suggested that a TBI can amplify pre-existing psychiatric disorders, although more research is needed [46]. This phenomenon, if true, further complicates the process of determining whether observed post-TBI neuropsychiatric sequelae are related to the injury.

Current research suggests that neurorehabilitation is beneficial to TBI patients although more research is needed to determine optimal intensities and durations of neurorehabilitation programs to be able to effectively treat different forms of TBI’s and their respective symptoms [47]. Intensive neurorehabilitation strategies including early trauma center rehabilitation have been shown to promote functional recovery and are therefore strongly recommended for severe TBI cases. Additionally, evidence supports the use of neurorehabilitation to restore deficits in memory, attention, executive functioning, and communication. Oberholzer and Müri conclude in their 2019 paper that approaches to the neurorehabilitation of TBI patients ought to be tailored to the particular sequelae of the patient in order to account for the heterogeneity among cases and that this form of rehabilitation can benefit significantly from the use of a collaborative team of clinicians, neuropsychologists, physical physiotherapists, speech therapists, and nurses [47]. One reason supporting the use of this approach is that the research needed to establish effective, standardized rehabilitation strategies is nearly impossible to conduct due to the ethical considerations of obtaining informed consent and the extended amount of time needed before the results of rehabilitation often take effect. 

Neuroinflammation is an important secondary response to TBI that provides both beneficial and detrimental effects to the recovery process. This inflammatory response which centers on glial cell activation results in an immediate neuroprotective defense which in excess can lead to further neurodegeneration and loss of function [48]. Multifaceted treatment of TBI patients should therefore involve drugs to mitigate the neurotoxic effects of overactive neuroinflammation while promoting its neurotrophic effects. While many drugs are currently being studied for their effectiveness in achieving these complex effects, statins have proven to be very promising in their ability to improve functional recovery in TBI patients [48].

MSA

MSA is a rare and progressive neurodegenerative disorder characterized by widespread brain atrophy, predominantly in the basal ganglia, brainstem, and cerebellum, as well as frontal and temporal lobes, though the cortex also shows imaging widespread atrophy [49]. This leads to a combination of autonomic dysfunction and motor symptoms. This dysfunction can manifest in various ways and can have a significant impact on mood and overall well-being common presentations include orthostatic hypotension, tachycardia or bradycardia, gastrointestinal issues (gastroparesis, constipation, or diarrhea), sweating abnormalities, temperature dysregulation, and bladder or bowel dysfunction. These symptoms can impact mood disturbances such as depression, anxiety, and social isolation [50].

This is of particular importance given the extremely high prevalence of depression and anxiety in patients who have MSA. Research has shown that more than half of all patients with MSA have depression and those who have moderate to severe depressive symptoms range anywhere from 10.4% to 46.3% based on the Beck Depression Inventory [51]. This high prevalence marks depression as the most consistent and impactful psychiatric manifestation of MSA compared to other neuropsychiatric disorders [51]. Other studies in China have also indicated that anxiety and depression tend to be comorbid psychiatric symptoms in patients with MSA given that 62.0% and 71.7% of patients were found to have at least mild depression and anxiety symptoms, respectively [52].

Interestingly, there also appears to be a correlation between gender and behavioral and cognitive manifestations of MSA. One study found that, at baseline, women with MSA had poorer global cognitive state and visuospatial abilities, and a higher prevalence of depression and apathy compared to male participants [53]. Additionally, while these gender differences need to be further explored, it seems that females with MSA tend to deteriorate more over time and have worsening depression and anxiety than men with MSA. While effective management and support, including medical treatment, lifestyle adjustments, and psychological counseling, can help individuals cope with MSA and mitigate its impact on their emotional well-being, more importance and insight should be given to early-intervention patients who have MSA to help mitigate depression and anxiety manifestations.

The role of alpha-synuclein pathology in MSA is a central aspect of its pathogenesis. In MSA, alpha-synuclein accumulates abnormally within certain brain structures, leading to neurodegeneration and the manifestation of various symptoms; both motor and psychiatric [54]. While still not well understood, some studies suggest that in MSA, A5 noradrenergic neurons are severely lost and this leads to alpha-synuclein accumulation and may contribute at least to the cardiovascular and respiratory manifestations commonly seen in these patients [55]. Motor manifestations of alpha-synuclein aggregation in patients with MSA can manifest in a multitude of ways. MSA-P (MSA with predominant parkinsonism) is one of the clinical subtypes of MSA characterized by prominent motor symptoms [56]. The accumulation of alpha-synuclein in the basal ganglia and other motor control regions disrupts normal motor function and can result in symptoms such as bradykinesia, rigidity, postural instability, dystonia, and resting tremors, which resemble those seen in Parkinson's disease [56]. Another subtype of MSA can present with predominant cerebellar ataxia [57]. Alpha-synuclein pathology in the cerebellum contributes to problems with coordination and balance which leads to symptoms of ataxia, gait disturbances, and dysarthria [57]. 

Regarding psychiatric symptoms, alpha-synuclein pathology in the emotional regulatory centers of the brain can potentially lead to the development of depression, anxiety, cognitive impairment, and, albeit rarely, psychosis in individuals with MSA. Growing evidence has shown that accumulation of alpha-synuclein in the raphe nuclei and efferent brain areas can lead to serotonergic dysfunction thereby leading to the pathogenesis of depression and anxiety in MSA patients [58]. While the exact mechanisms by which alpha-synuclein pathology leads to cognitive impairment in MSA are not fully understood, there is growing evidence that the accumulation of alpha-synuclein in hippocampal oligodendrocytes might be the cause [59]. Some MSA patients can develop psychosis although it is less common than in other neurodegenerative disorders like Parkinson's disease [59,60]. While the presence of alpha-synuclein aggregates in brain regions is likely responsible for the sensory and perceptual processing that contribute to these symptoms, intermediate CAG repeats within the *ATXN2 *gene in individuals with MSA can increase the risk of developing psychosis-related symptoms [60].

It's important to note that the specific distribution and severity of alpha-synuclein pathology can vary between MSA patients, leading to differences in the expression of motor and psychiatric symptoms. Additionally, the overall burden of dealing with a progressive neurodegenerative disease can exacerbate psychiatric issues. Moreover, the exact mechanisms through which alpha-synuclein aggregates lead to these symptoms are not fully elucidated and remain an active area of research. Currently, there is no cure for MSA, and treatment primarily focuses on partial and transient relief of symptoms in patients [54,56]. A better understanding of the role of alpha-synuclein pathology in MSA is critical for the development of potential disease-modifying therapies in the future.

Ethical considerations in treating psychiatric symptoms in neurological disease

In clinical practice, neuropsychiatrists play a crucial role in assessing and managing psychiatric symptoms associated with various neurological disorders, ranging from seizure disorders to central nervous system degenerative diseases [61]. Their work demands flexibility as they integrate knowledge from multiple disciplines such as general psychiatry, neuropsychology, neuroanatomy, and neurophysiology, while also keeping abreast of emerging neuroscience research [61].

Aside from clinical skills, neuropsychiatrists grapple with complex ethical issues inherent in treating patients with neuropsychiatric conditions [61]. These challenges encompass safeguarding patient autonomy, addressing caregiver neglect, and respectfully working with individuals with functional neurologic conditions. Moreover, as advancements in prevention, diagnostics, and treatment emerge, novel ethical dilemmas continue to surface, necessitating a profound understanding of fundamental ethical principles in psychiatric care [61]. It remains paramount for clinicians to strive for a balance between treatment objectives and enhancing the quality of life for their patients.

The growing demand for innovative mental health treatments has spurred interest in therapeutic agents like psychedelics, ketamine, and neuromodulatory technologies [62]. Despite their potential, ethical quandaries arise concerning issues such as informed consent, the impact of expectancy on clinical response, and distributive justice. These treatments are pursued as alternatives to traditional antidepressants due to their limitations, the increasing prevalence of mental health issues, and advancements in brain research. However, caution is warranted due to the multifaceted challenges spanning psychological, physiological, political, cultural, and historical dimensions. Ethical considerations, particularly at the intersection of clinical and research ethics, add further complexity to the utilization of these treatments [62].

Obtaining informed consent from patients with progressive neurological diseases, such as ALS, Parkinson’s disease, and AD, presents distinct challenges due to the conditions' impact on cognitive abilities, decision-making capacity, and communication skills. It is also important to consider guardianship issues and who would be responsible for making a life-altering decision in these individuals' lives. Addressing these challenges necessitates a patient-centered and adaptable approach to the informed consent process. Healthcare professionals may need to employ alternative communication methods, involve interdisciplinary teams, and establish mechanisms for ongoing communication with patients and their families, while maintaining and considering the patient’s right to privacy. Furthermore, careful navigation of legal and ethical considerations is essential to safeguard the rights and well-being of patients with progressive neurological diseases [62].

Psychiatric and neurological care and research encounter complexities in obtaining valid consent due to potential cognitive interference and individual capacity assessments. Novel treatments also require additional disclosures, such as financial implications and ongoing care access [61,62]. Despite the significance of informed consent, participation criteria for rapid treatments may inadvertently lower thresholds, driven by hopes for "miracle cures." Patient-reported outcomes are further complicated by outcome expectancies, particularly in trials of innovative treatments, underscoring the necessity for comprehensive long-term therapeutic plans [61,62].

Challenges associated with fast-acting therapies altering consciousness include unknown mechanisms and ethical concerns in blinded trials [62,63]. Strategies to address these challenges may involve alternative trial designs and deliberate ambiguity during the consent process regarding acute effects, albeit with raised ethical and logistical considerations. Bioethical analysis highlights individual-level considerations like consent capacity while also acknowledging community and structural challenges, especially concerning distributive justice in psychiatric care and research. A narrow focus on socioeconomic vulnerability risks excluding certain groups from crucial research, perpetuating historical inequities, and overlooking marginalized needs [62,63].

Future challenges in implementing new brain-based treatments encompass equity and access issues, with patients encountering barriers such as limited resources and time constraints. Overcoming these hurdles necessitates prioritizing diverse patient and caregiver input and utilizing translated, culturally adapted, and locally validated screening tools in clinical trials and treatment implementation.

Implications and future directions

The intricate relationship between neurological diseases and psychiatric manifestations underscores the complexity of the human brain and the profound impact these conditions have on individuals' lives. As we have explored in this review, conditions such as AD, FTD, Parkinson's disease, MS, stroke, epilepsy, Huntington's disease, ALS, TBI, and MSA are not merely characterized by their primary neurological symptoms but are also accompanied by a myriad of psychiatric manifestations. Each disorder exhibits a unique array of neuropsychiatric manifestations, further complicating diagnosis and treatment.

AD, for instance, not only ravages cognitive functions but also induces emotional distress, personality changes, agitation, and psychosis, making it challenging for patients and caregivers alike. Similarly, FTD presents with behavioral symptoms that can lead to sociopathic behavior, posing ethical dilemmas in legal contexts. Parkinson's disease, on the other hand, not only impairs motor function but also predisposes individuals to depression, anxiety, apathy, psychosis, and impulse control disorders, significantly impacting their quality of life.

In MS, the interplay between physical symptoms, cognitive dysfunction, and emotional disturbances contributes to the high prevalence of psychiatric conditions, particularly depression and anxiety, in affected individuals. Meanwhile, stroke survivors grapple with post-stroke depression and anxiety, exacerbating the challenges posed by physical impairments and communication deficits. Epilepsy patients experience a spectrum of psychiatric comorbidities, with mood disorders, anxiety, and psychosis often intertwined with seizure activity.

Understanding the interplay between these neurological and psychiatric symptoms is paramount for providing comprehensive care to patients. By fostering collaboration, innovation, and a steadfast commitment to ethical principles, we can strive towards better outcomes and improved quality of life for individuals affected by these challenging conditions. Looking ahead, the integration of novel therapeutic modalities, such as psychedelics and neuromodulatory technologies, as well as, recent advancements in understanding the underlying neurobiology of these disorders have shed light on potential treatment avenues. These efforts can hopefully aid in alleviating the burden of neuropsychiatric symptoms on patients and their caregivers. As the field continues to evolve, it is imperative to prioritize patient well-being, uphold ethical principles, and strive for inclusivity and equity in the delivery of neurological and psychiatric care.

As we look to the future, continued research efforts are crucial for unraveling the complexities of the neurological-psychiatric interface. By fostering interdisciplinary collaboration and embracing a holistic approach to patient care, we can pave the way for advancements in diagnosis, treatment, and support services, ultimately enhancing the lives of individuals affected by these challenging conditions.

## Conclusions

Neurological diseases like Alzheimer's, FTD, Parkinson's, MS, stroke, epilepsy, Huntington's, ALS, TBI, and MSA are not only characterized by neurological symptoms but also by various psychiatric manifestations, complicating diagnosis and treatment. For instance, Alzheimer's induces cognitive decline and emotional distress, Parkinson's leads to motor impairments and mood disorders, and MS intertwines physical symptoms with emotional disturbances. Understanding these complex relationships is crucial for comprehensive care. Collaboration, innovation, and ethical commitment are essential for improving outcomes. New therapeutic approaches and continued research will advance diagnosis, treatment, and support, enhancing the lives of those affected.
